# A Systematic Review and Meta-Analysis Examining Whether Changing Ovarian Sex Steroid Hormone Levels Influence Cerebrovascular Function

**DOI:** 10.3389/fphys.2021.687591

**Published:** 2021-06-17

**Authors:** Bethany D. Skinner, Rebecca J. Davies, Samuel R. Weaver, N. Tim Cable, Samuel J. E. Lucas, Rebekah A. I. Lucas

**Affiliations:** ^1^School of Sport, Exercise and Rehabilitation Sciences, College of Life and Environmental Sciences, University of Birmingham, Birmingham, United Kingdom; ^2^Centre for Human Brain Health, University of Birmingham, Birmingham, United Kingdom

**Keywords:** cerebrovascular function, systematic review, meta-analysis, ovarian sex steroid hormones, cerebral blood flow

## Abstract

Sex differences in cerebrovascular disease rates indicate a possible role for ovarian sex steroid hormones in cerebrovascular function. To synthesise and identify knowledge gaps, a systematic review and meta-analysis was conducted to assess how ovarian sex steroid hormone changes across the lifespan affect cerebrovascular function in women. Three databases (EMBASE, MEDLINE and Web of Science) were systematically searched for studies on adult cerebrovascular function and ovarian sex steroid hormones. Forty-five studies met pre-defined inclusion criteria. Studied hormone groups included hormone replacement therapy (HRT; *n* = 17), pregnancy (*n* = 12), menstrual cycle (*n* = 7), menopause (*n* = 5), oral contraception (*n* = 2), and ovarian hyperstimulation (*n* = 2). Outcome measures included pulsatility index (PI), cerebral blood flow/velocity (CBF), resistance index (RI), cerebral autoregulation, and cerebrovascular reactivity. Meta-analysis was carried out on HRT studies. PI significantly decreased [−0.05, 95% CI: (−0.10, −0.01); *p* = 0.01] in post-menopausal women undergoing HRT compared to post-menopausal women who were not, though there was considerable heterogeneity (*I*^2^ = 96.8%). No effects of HRT were seen in CBF (*p* = 0.24) or RI (*p* = 0.77). This review indicates that HRT improves PI in post-menopausal women. However, there remains insufficient evidence to determine how changing ovarian sex steroid hormone levels affects cerebrovascular function in women during other hormonal phases (e.g., pregnancy, oral contraception).

## Introduction

Sex differences in the rate and occurrence of cerebrovascular diseases (i.e., stroke and vascular dementia) indicate a possible role for ovarian sex steroid hormones in brain vascular function and regulation. For example, despite women having a lower risk of stroke than men during midlife their risk doubles in the decade after menopause (Lisabeth and Bushnell, 2012), a time in which endogenous oestrogen and progesterone concentrations decline significantly.

Prolonged exposure to oestrogen has been shown to promote vasodilatory factors (Skarsgard et al., 1997; Geary et al., 2000; Pelligrino et al., 2000). Additionally, oestrogen has demonstrated a number of neuroprotective effects including suppression of the inflammatory response and improved perfusion after ischaemic injury (Hurn et al., 1995; Santizo et al., 2002). The effects of progesterone are far less clear, with the literature suggesting that it both promotes and reduces the inflammatory response to cerebrovascular injury (Gibson et al., 2005; Sunday et al., 2006).

There is substantial evidence from cellular and animal experimental models that gonadal sex hormones directly affect brain vascular function. Indeed, narrative reviews by Orshal and Khalil (2004) and Krause et al. (2006) examine the effect of oestrogen, progesterone, and testosterone on vascular tone and the cerebrovasculature. To date, however, no review (systematic or otherwise) has examined the effects of changing ovarian sex steroid hormones on cerebrovascular function in humans. Subsequently, it remains unclear how the interaction, absence or increase in oestrogen and progesterone affects cerebrovascular function in women.

Therefore, to synthesise and identify knowledge gaps a systematic review and meta-analysis was conducted to assess how changes in ovarian sex steroid hormones across the lifespan affect cerebrovascular function in women. This will help inform future research directions relating to sex hormones and cerebrovascular function, as well as sex differences in cerebrovascular diseases.

## Methods

This review utilised a systematic search strategy to provide a succinct yet thorough overview of the available literature. The protocol used was informed by the Preferred Reporting Items for Systematic Reviews and Meta Analyses (PRISMA-P) guidelines (Shamseer et al., 2015).

### Search Strategy

A formal literature search, using bibliographic search databases, was the primary method of identifying relevant texts. The electronic databases MEDLINE, Web of Science and EMBASE were searched for publications relating to ovarian sex steroid hormones and cerebrovascular function using MeSH terms and free-text terms to capture relevant research. Core keywords used in the search included for example, “cerebrovascular circulation” OR “middle cerebral artery” OR “brain blood flow” AND “gonadal steroid hormones” OR “menstrual cycle.” The complete search strategy can be found in Appendix 1. A manual search for work on ovarian sex steroid hormones and cerebrovascular function, including article reference lists, was conducted to ensure all relevant texts were identified. Database and manual searches included texts from the first available date to March 2021.

### Inclusion and Exclusion Criteria

Inclusion and exclusion criteria were developed through researcher discussion and guided by the Population, Intervention, Comparison/Control Group, Outcome, and Time (PICOT) framework (Higgins and Green, 2011). The criteria covered population (e.g., postmenopausal women), intervention/domain studied (e.g., hormone replacement therapy), study type (e.g., randomised control trials), and relevance/outcome measures (e.g., brain blood flow). Research articles needed to address both cerebrovascular function and changing ovarian sex steroid hormones within a healthy adult population (>18 years) to be suitable for inclusion. Exclusion criteria included clinical populations (i.e., cerebrovascular injury, pre-eclampsia), synthetic hormone treatment, animal studies, as well as sources of information such as book chapters, conference abstracts or poster formats.

### Study Selection

Two reviewers (BDS, RJD) independently searched the literature using three databases and the defined keywords. All citations identified in the search were independently screened by the reviewers on the basis of the title and the abstract to assess their match with inclusion criteria. To ensure the inclusion/exclusion criteria were applied consistently, 20% of identified citations were screened by both reviewers and the results compared. Disagreements regarding eligibility of studies were resolved by discussion and consensus with a third reviewer (RAIL). The reviewers checked the references of included studies to identify any relevant papers not captured in the search. Included title and abstracts were then screened for their full texts.

### Data Extraction and Quality Assessment

Full texts deemed eligible for inclusion underwent data extraction using a data extraction form. This included participant demographics (e.g., number, age, population), study characteristics (e.g., design, protocol), outcome measures (e.g., hormones reported, brain blood flow measures) and results. Where possible, results were expressed as absolute values (mean ± SD) in order to determine percentage change across time points/experimental groups. To minimise bias, and ensure the accuracy of the study selection procedure, a random sample of 20% of included studies were extracted by both reviewers and results compared. The two independent reviewers graded all identified studies as either a high (level 1; e.g., randomised control trial), moderate (level 2; e.g., cohort, case-control studies) or low (level 3; e.g., cross-sectional study) level of evidence. The criterion for each level of evidence can be found in Appendix 2.

Reviewers applied a modified version of the National Heart, Lung, and Blood Institute quality assessment tool for observational cohort and cross-sectional studies (National Heart, Lung, and Blood Institute, 2014) to assess the internal validity and risk of bias for each study. They independently evaluated the 15 components of the tool as “Yes,” “No,” “Not Applicable” or “Not Recorded” to achieve a rating of “High,” “Moderate” or “Low” to assess the quality of included studies. In case of disagreement the third reviewer was consulted and a consensus opinion reached.

### Data Synthesis and Analysis

A “low hormone phase” and “high hormone phase” were identified (defined in Table 1) to allow for comparisons between studies within the same hormone group. The absolute and relative change in cerebrovascular function was calculated from a low to high hormone phase and summarised separately within each hormone group (e.g., menopause, pregnancy). Specifically, the markers of cerebrovascular function were pulsatility index (PI; marker of downstream flow resistance), cerebral blood flow/velocity (CBF; marker of cerebral perfusion), resistance index (RI; marker of downstream flow resistance), cerebrovascular reactivity to CO_2_ (CVR; the vasodilatory reserve capacity to a CO_2_ stimulus), and cerebral autoregulation (CA; the capacity to maintain cerebral blood flow despite changes in perfusion pressure). If measures were presented as mean difference with 95% confidence intervals, then standard deviation was calculated using the method described in the Cochrane Handbook (7.7.7.2; Higgins and Green, 2011). Due to the wide heterogeneity of included studies in terms of study design, population and outcome measures, only some hormone groups were deemed to have sufficient data for a meta-analysis. This was determined by there being 4 or more studies reporting the outcome measure in a comparable manner, reporting data as mean and standard deviation, and the low and high hormone group being clearly defined. As such, only HRT studies reporting pulsatility index, cerebral blood flow/velocity and resistance index were included in the meta-analysis to quantify the differences between high and low hormone groups.

**Table 1:** Definition of “low hormone phase” and “high hormone phase” for each hormone group.

**Hormone group**	**Low hormone phase**	**High hormone phase**
Pregnancy	Non-pregnant	2nd−3rd trimester
Menstrual cycle	Follicular phase	Ovulation or luteal phase
Menopause	Post-menopause	Pre-menopause
Hormone Replacement Therapy (HRT)	Pre-HRT or control group	HRT
Ovarian hyperstimulation	Pituitary suppression	Human menopausal gonadotropin (hMG) stimulation
Oral Contraception (OC)	Non-OC users	OC Users

Hormone group differences were investigated using a random effect model for outcome measures of PI, CBF, and RI. Mean differences between a low and high hormone phase were calculated, and overall effect estimates were calculated using random effect models, and reported alongside estimate significance (*p*-value) and heterogeneity (*I*^2^). The *I*^2^ statistic was examined to evaluate heterogeneity, with *I*^2^ > 50% and *I*^2^ > 75% indicative of substantial and considerable heterogeneity, respectively (Higgins et al., 2003).

For studies reporting PI, moderator analysis was carried out to identify possible sources of heterogeneity, by comparing overall and subgroup estimated effects based on the artery in which measures were taken and the type/types of medication used. There was an insufficient number of studies to perform sub-group analyses for CBF and RI. Meta-regression were conducted using mixed effects models with each of these factors included as a moderator, to determine their impact on both effect size and heterogeneity in each outcome measure. The impact of moderators on the hormone level effect was evaluated by the proportion of heterogeneity accounted for, with the significance of this assessed by omnibus tests for the overall model effect and Wald-type Chi-Squared tests for each moderator within the model.

Potential outliers were identified and evaluated by externally standardised studentized deleted residuals, based on the size of each study's individual residual, with values < -2 or >2 considered outlying. The impact of each study on the overall effect was then assessed using model fit impact analysis (DFFITS and Cook's distance); covariance from the mean; residual heterogeneity test statistics; overall result influence (hat values), and study weight (Viechtbauer, 2010). If a study was deemed to be outlying, the random effects model in which it was included was refitted excluding the outlier and both model fits reported in the final results. All statistical analyses were carried out in RStudio (R Core Team, 2019) with meta-analysis conducted using the Metafor package (Viechtbauer, 2010). All effect estimates are reported as mean difference with 95% confidence intervals (MD, [95% CI]), unless otherwise stated, with statistical significance of *p*-values being assessed at α = 0.05.

## Results

### Included Studies

**Figure 1:**
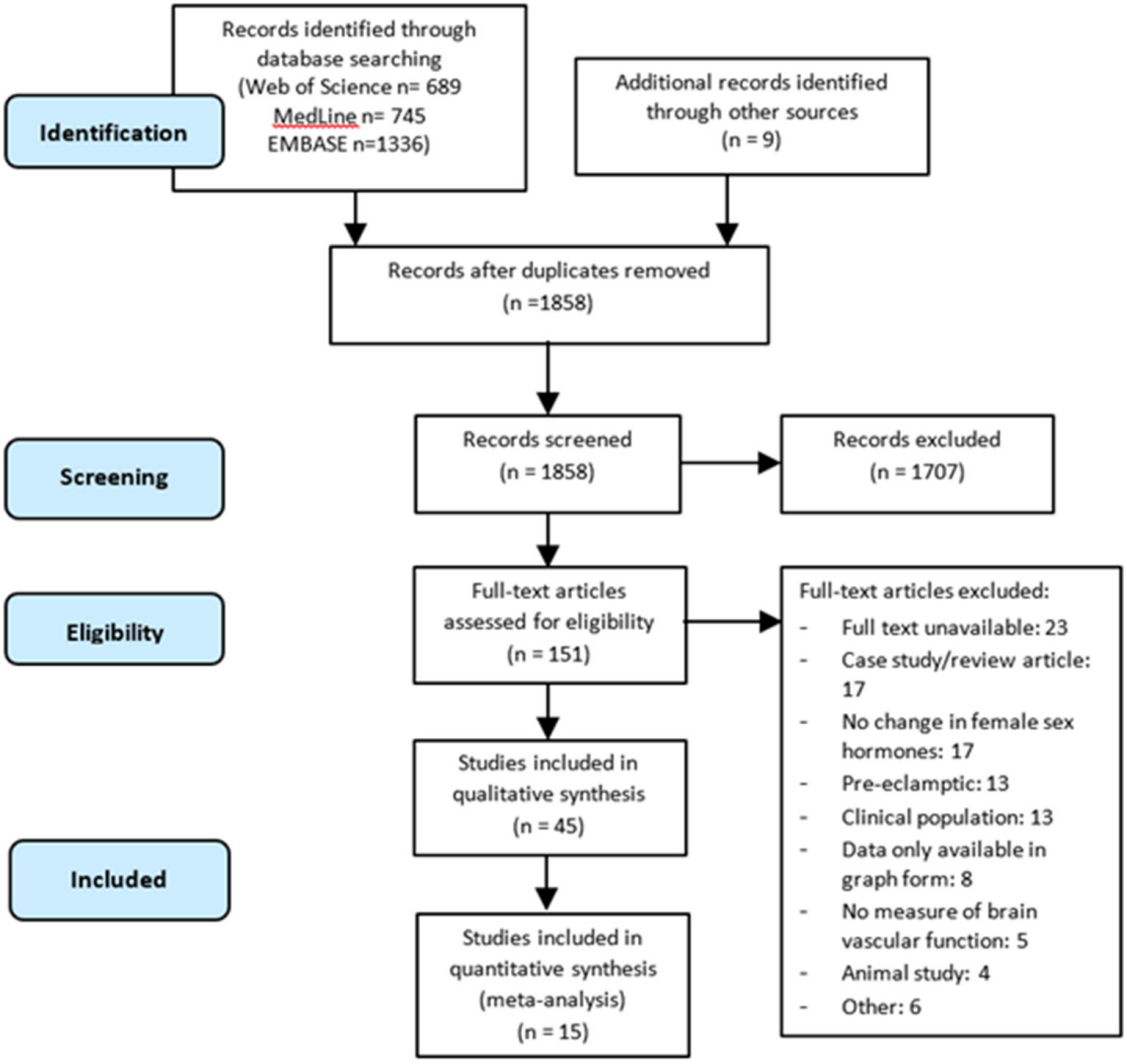
PRISMA-P flow diagram of the literature search and selection process for articles included in the systematic review and meta-analysis.

A total of 2,770 citations were identified through the electronic database search, and an additional nine records were identified through manual searches. Once duplicates were removed, the title and abstracts of 1,858 articles were screened, leaving 151 full texts to be assessed for eligibility. Overall, 45 articles met the inclusion criteria for the qualitative synthesis. Of the 45 articles, 15 articles were suitable for inclusion in the meta-analyses (Figure 1).

### Study Characteristics

**Figure 2:**
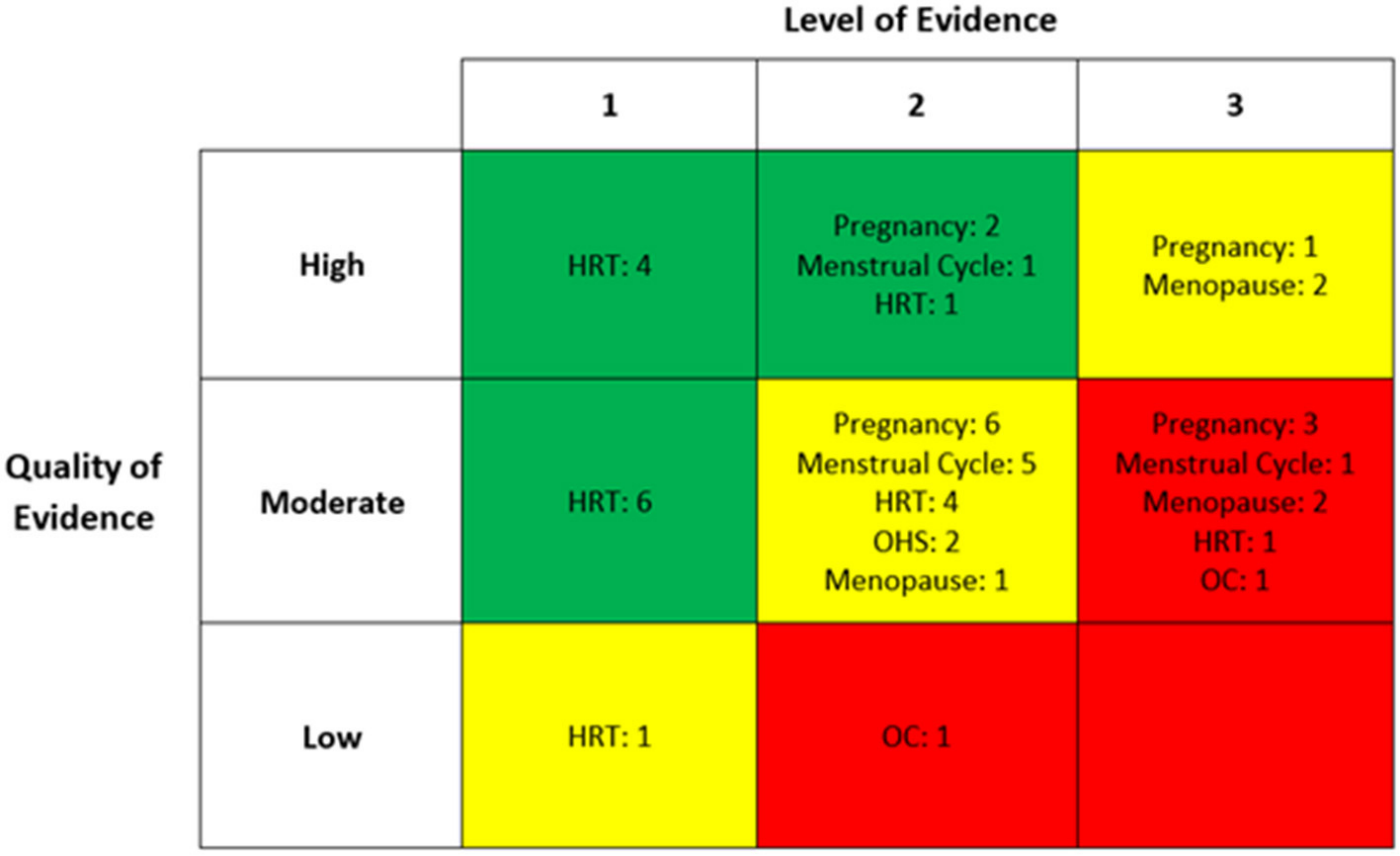
The level and quality of evidence of each included study within individual hormone group. HRT, hormone replacement therapy; OHS, ovarian hyperstimulation; OC, oral contraception.

Of the studies included in the quantitative synthesis, 29 were based in Europe, eight in North America, five in Asia, and three in South America. Six of these were randomised controlled trials, 15 were non-randomised controlled trials or cohort studies, 14 were cross-sectional, nine were longitudinal and one was an observational study. Hormone replacement therapy (HRT) was the most frequently studied hormone group (*n* = 17), followed by pregnancy (*n* = 12), menstrual cycle (*n* = 7), menopause (*n* = 5), oral contraception (*n* = 2), and ovarian hyperstimulation (*n* = 2). Eleven studies were assessed to be of high quality and therefore, low risk of bias. Thirty-two studies were deemed to be of moderate quality and two of low quality. Figure 2 summarises both the level and quality of evidence of included studies, both overall and within hormone groups.

### Effect of Changing Ovarian Sex Steroid Hormones on Pulsatility Index

Twenty-six studies were identified that reported PI in low and high hormone phases (Table 2). PI was most frequently reported using the internal carotid artery (ICA; *n* = 22), with fewer studies reporting PI of the middle cerebral artery (MCA; *n* = 13), external carotid artery (ECA; *n* = 4), common carotid artery (CCA; *n* = 4), and posterior cerebral artery (PCA; *n* = 1).

**Table 2:** Characteristics for included studies reporting Pulsatility index (PI; arbitrary units).

**Study**	**Technique**	**Hormone group**	**Sample size (n; low hormone phase, high hormone phase)**	**Vessel insonated**	**Outcome measure**	**Low hormone phase; mean (SD)**	**High hormone phase; mean (SD)**	**% change in PI from low to high hormone phase**
**Cagnacci et al. (****2000****)**	Vascular Doppler Ultrasound	HRT	13, 18	ICA	PI	1.15 (0.3)	1.09 (0.19)	−5
**Cacciatore et al. (****1998****)**	Vascular Doppler Ultrasound	HRT	58	ICA	PI	0.98 (0.08)	0.85 (0.08)	−13
Crook et al. (1991)^‡^	Vascular Doppler Ultrasound	HRT	12	ICA	PI	0.92 (0.77–1.18)	0.82 (0.70–0.90)	−11
**Darj et al. (****1999****)**	Vascular Doppler	HRT	20	CCA	PI	1.75 (0.49)	1.66 (0.37)	−5
	Ultrasound			ECA	PI	2.04 (0.7)	2.02 (0.53)	−1
				ICA	PI	1.05 (0.27)	1.13 (0.31)	8
**Guvenal et al. (****2009****)**	Transcranial Doppler Ultrasound	HRT	47	MCA ICA	PI PI	0.65 (0.08) 0.64 (0.05)	0.62 (0.05) 0.63 (0.06)	−5 −2
**Huang et al. (****2009****)**	Vascular Doppler Ultrasound	HRT	20	ICA	PI	0.96 (0.15)	1.13 (0.35)	18
Jackson and Vyas (1998)	Vascular Doppler Ultrasound	HRT	15	ICA	PI	1.07 (0.28)	0.96 (0.17)	−11
**Lazar Jr et al. (****2004****)**	Vascular Doppler Ultrasound	HRT	38	ICA	PI	1.08 (0.28)	0.99 (0.22)	−7
**Naessen and Bakos (****2001****)**	Vascular Doppler Ultrasound	HRT	18	ICA CCA	PI PI	1.36 (0.34) 2.03 (0.52)	1.32 (0.26) 1.78 (0.36)	−3 −12
				ECA	PI	2.26 (0.83)	2.07 (0.48)	−8
**Pan et al. (****2002****)**	Vascular Doppler Ultrasound	HRT	40	MCA CCA	PI PI	0.87 (0.1) 1.30 (0.17)	0.92 (0.1) 1.35 (0.22)	6 3
				ICA	PI	0.97 (0.19)	0.94 (0.14)	−3
**Penotti et al. (****1996a****)**	Vascular Doppler Ultrasound	HRT	23	MCA ICA	PI PI	0.73 (0.07) 0.73 (0.07)	0.71 (0.08) 0.82 (0.07)	−2 12
**Penotti et al. (****1998****)**	Vascular Doppler Ultrasound	HRT	30	MCA	PI	0.81 (0.01)	0.66 (0.01)	−19
				ICA	PI	0.81 (0.01)	0.65 (0.02)	−20
**Persico et al. (****2005****)**	Vascular Doppler Ultrasound	HRT	14	ICA	PI	1.40 (0.20)	1.05 (0.01)	−25
**Wender et al. (****2011****)**	Vascular Doppler Ultrasound	HRT	75	ICA	PI	0.90 (0.16)	0.84 (0.15)	−7
Brackley et al. (1998)^‡^	Transcranial Doppler Ultrasound	Pregnancy	17	MCA	PI	0.73 (0.64–0.78)	0.85 (0.81–0.96)	16
	Vascular Doppler Ultrasound	Pregnancy	17	ICA	PI	0.83 (0.80–0.96)	0.98 (0.87–1.08)	18
Janzarik et al. (2014)	Transcranial Doppler Ultrasound	Pregnancy	26, 61	MCA	PI	0.66 (0.10)	0.84 (0.14)	27
			26, 59	PCA	PI	0.59 (0.08)	0.78 (0.14)	32
Lindqvist et al. (2006)	Transcranial Doppler Ultrasound	Pregnancy	14	MCA	PI	0.79 (0.16)	0.99 (0.22)	25
Serra-Serra et al. (1997)	Transcranial Doppler Ultrasound	Pregnancy	25, 22	MCA	PI	0.82 (0.14)	0.91 (0.14)	11
Brackley et al. (1999)^‡^	Transcranial Doppler Ultrasound	Menstrual Cycle	19	MCA	PI	0.72 (0.70–0.82)	0.81 (0.71–0.94)	13
	Vascular Doppler	Menstrual Cycle	19	ICA	PI	0.87 (0.76 – 0.98)	0.90 (0.81 – 1.01)	4
	Ultrasound			ECA	PI	2.52 (2.31–3.30)	2.48 (2.31–2.99)	−2
Hazlett and Edgell (2018)	Transcranial Doppler Ultrasound	Menstrual Cycle	14	MCA	PI	0.76 (0.04)	0.77 (0.03)	1
Krejza et al. (2004)^•^	Vascular Doppler	Menstrual Cycle	14	ICA	PI	0.99 (1.05)	1.03 (0.99)	4
	Ultrasound			CCA	PI	1.79 (1.63)	1.84 (1.84)	3
				ECA	PI	2.13 (2.06)	2.34 (2.29)	10
Arangino et al. (1998)	Vascular Doppler Ultrasound	Oral Contraception	22, 22	ICA	PI	1.30 (0.12)	1.46 (0.19)	12
		Menstrual Cycle	10, 12	ICA	PI	1.32 (0.13)	1.27 (0.19)	−4
Cagnacci et al. (1999)	Vascular Doppler Ultrasound	Oral Contraception	17, 17	ICA	PI	1.34 (0.12)	1.54 (0.17)	15
		Menstrual Cycle	18	ICA	PI	1.34 (0.12)	1.30 (0.16)	−3
Penotti et al. (1996b)	Vascular Doppler	Menopause	18, 18	MCA	PI	0.80 (0.11)	0.68 (0.15)	−15
	Ultrasound			ICA	PI	0.79 (0.1)	0.68 (0.15)	−14
Robertson et al. (2008)	Transcranial Doppler Ultrasound	Menopause	12, 12	MCA	PI	0.81 (0.12)	0.86 (0.18)	6
Shamma et al. (1992)	Transcranial Doppler Ultrasound	Ovarian Hyper-stimulation	9	MCA	PI	0.72 (0.08)	0.82 (0.04)	14

‡ *Median (Inter-quartile range)*

• *Mean (median). Studies in **bold** indicate those included in the meta-analyses. HRT, hormone replacement therapy; ICA, internal carotid artery; CCA, common carotid artery; ECA, external carotid artery; MCA, middle cerebral artery; PCA, posterior cerebral artery*.

**Figure 3:**
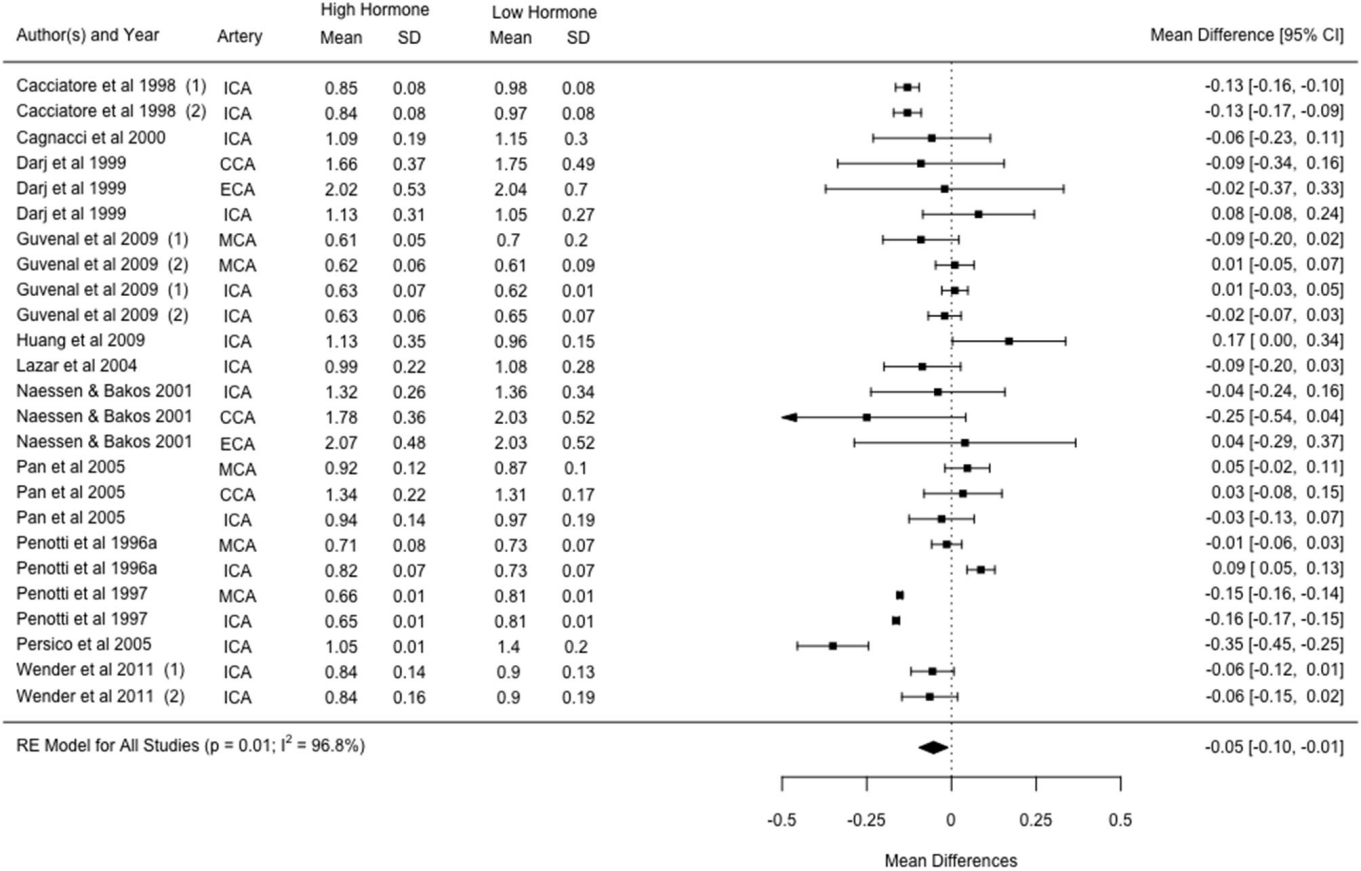
Forest plot showing mean difference and 95% confidence intervals for the impact of HRT administration in post-menopausal women compared to a post-menopausal control group on the pulsatility index. Numbers indicate different cohorts within a study (i.e., different HRT types). ICA, internal carotid artery; CCA, common carotid artery; ECA, external carotid artery; MCA, middle cerebral artery.

Fourteen studies investigated changes with HRT and showed that PI was 4.6 ± 10.2% lower in the high hormone phase as compared to the low phase. Twelve studies reporting PI changes with HRT were suitable for inclusion in the meta-analysis. Within these 12 studies, three reported outcomes in two experimental groups (i.e., different HRT treatments), and six reported outcomes in two or more vessels, resulting in a total of 25 cohorts. The combined effects of the 25 cohorts showed an estimated mean difference of −0.05 [−0.10, −0.01] from a low to high hormone phase, which was significant and showed considerable levels of heterogeneity (*p* = 0.01; *I*^2^ = 96.8%; Figure 3). Mixed effect modelling was used to evaluate the effects of variation in HRT type and vessel of insonation, however little-to-no heterogeneity was accounted for by either moderator (5.96%, *p* = 0.303; and 0%, *p* = 0.952, respectively).

Four studies examined changes in PI with pregnancy, showing a 22 ± 8% higher PI in a high hormone phase compared to a low hormone phase. Five studies examining changes across the menstrual cycle, showed PI was 3 ± 6% higher in the high hormone phase compared to the low hormone phase, while two studies showed PI to be 8 ± 12% lower post-menopause compared to pre-menopause. PI increased by 14 ± 2% in the high hormone phase compared to the low hormone phase in oral contraceptive use (*n* = 2), and increased by 14% in the one study observing changes in PI from a low to high hormone phase in ovarian hyperstimulation.

### Effect of Changing Ovarian Sex Steroid Hormones on Cerebral Blood Flow/Velocity

A total of 25 included studies reported an index of CBF, the characteristics of which are summarised in Table 3. All but two studies presented CBF as indexed by blood flow velocity through a vessel. CBF was most frequently reported using the MCA (*n* = 14), with fewer studies reporting CBF in the ICA (*n* = 12), PCA (*n* = 2), CCA (*n* = 2), ECA (*n* = 1), and vertebral artery (VA; *n* = 1).

**Table 3:** Characteristics for included studies reporting cerebral blood flow/velocity (CBF).

**Study**	**Technique**	**Hormone group**	**Sample size (n; low hormone phase, high hormone phase)**	**Vessel insonated**	**Outcome measure (units)**	**Low hormone phase**	**High hormone phase**	**% change in CBF from low to high hormone phase**
Bergersen et al. (2006)^†^	Vascular Doppler Ultrasound	Pregnancy	14	ICA	Mean flow velocity (cm/s)	27 (5)	19 (6)	−30
Janzarik et al. (2014)	Transcranial	Pregnancy	26, 61	MCA	Mean flow velocity (cm/s)	61 (9)	57 (10)	−7
	Doppler			PCA	Mean flow velocity (cm/s)	41 (5)	38 (13)	−8
Lindqvist et al. (2006)	Transcranial Doppler	Pregnancy	13, 12	MCA	Mean flow velocity (cm/s)	57 (15)	57 (16)	0
Nevo et al. (2010)	Dual-beam, angle-independent, Doppler	Pregnancy	15, 129	ICA	Cerebral blood flow (mL/min)	294 (53)	382 (50)	23
Serra-Serra et al. (1997)	Transcranial Doppler	Pregnancy	25, 22	MCA	Mean flow velocity (cm/s)	70 (9)	60 (9)	−15
Sherman et al. (2002)	Transcranial Doppler	Pregnancy	30, 30	MCA	Mean flow velocity (cm/s)	69 (14)	57 (12)	−18
van Veen et al. (2016)	Transcranial Doppler	Pregnancy	18, 50	MCA	Mean flow velocity (cm/s)	72 (11)	66 (9)	−9
Zeeman et al. (2003)	Phase contrast magnetic	Pregnancy	9	MCA	Cerebral blood flow (mL/min)	148 (5)	118 (5)	−20
	resonance imaging			PCA	Cerebral blood flow (mL/min)	56 (3)	44 (2)	−21
**Acar et al. (****2005****)**	Vascular Doppler	HRT	18	ICA	Peak systolic velocity (cm/s)	59 (17)	62 (16)	5
	Ultrasound			VA	Peak systolic velocity (cm/s)	46 (14)	40 (8)	−13
**Cagnacci et al. (****2000****)**	Vascular Doppler Ultrasound	HRT	18, 13	ICA	Peak systolic velocity (cm/s)	34 (6)	40 (9)	18
**Clapauch et al. (****2007****)**	Vascular Doppler Ultrasound	HRT	9	ICA	Systolic flow velocity (cm/s)	52 (3)	54 (2)	4
**Guvenal et al. (****2009**)^*^	Transcranial	HRT	47	MCA	Peak systolic velocity (cm/s)	58 (14)	53 (13)	−8
	Doppler			ICA	Peak systolic velocity (cm/s)	39 (7)	36 (5)	−8
**Vidovic et al. (****2001****)**	Vascular Doppler Ultrasound	HRT	32	CCA	Peak systolic velocity (cm/s)	85 (17)	72 (15)	−15
Diomedi et al. (2001)	Transcranial Doppler	Menstrual cycle	20	MCA	Mean flow velocity (cm/s)	68 (13)	67 (12)	−1
Favre and Serrador (2019)	Transcranial	Menstrual cycle	13	MCA	Mean flow velocity (cm/s)	82 (16)	78 (19)	−5
	Doppler			ACA	Mean flow velocity (cm/s)	58 (15)	54 (10)	−6
Hazlett and Edgell (2018)	Transcranial Doppler	Menstrual cycle	14	MCA	Mean flow velocity (cm/s)	69 (4)	71 (3)	4
Krejza et al. (2001)	Vascular Doppler	Menstrual cycle	14	ICA	Mean flow velocity (cm/s)	42 (5)	47 (6)	12
	Ultrasound			CCA	Mean flow velocity (cm/s)	42 (4)	42 (5)	0
				ECA	Mean flow velocity (cm/s)	28 (5)	25 (5)	−11
Brislane et al. (2020)	Transcranial Doppler	Menopause	50, 50	MCA	Mean flow velocity (cm/s)	61 (15)	72 (15)	18
Iwamoto et al. (2021)	Vascular Doppler	Menopause	11, 10	ICA	Mean flow velocity (cm/min)	28 (8)	43 (10)	54
	Ultrasound				Blood flow (mL/min)	281 (106)	404 (87)	44
Matteis et al. (1998)	Transcranial Doppler	Menopause	40, 45	MCA	Mean flow velocity (cm/s)	60 (9)	62 (12)	4
Robertson et al. (2008)	Transcranial Doppler	Menopause	12, 12	MCA	Mean flow velocity (cm/s)	70 (10)	67 (11)	−4
Nevo et al. (2007)	Vascular Doppler Ultrasound	Ovarian hyperstimulation	12	ICA	Mean flow velocity (cm/s)	40 (2)	47 (2)	17
Shamma et al. (1992)	Transcranial Doppler	Ovarian hyperstimulation	9	MCA	Peak systolic velocity (cm/s)	98 (12)	105 (12)	7
Arangino et al. (1998)	Vascular Doppler Ultrasound	Oral contraceptive	22, 22	ICA	Peak flow velocity (cm/s)	51 (4)	45 (4)	−13
Cagnacci et al. (1999)	Vascular Doppler	Oral contraceptive	18, 17	ICA	Peak flow velocity (cm/s)	61 (7)	50 (2)	−18
	Ultrasound	Menstrual cycle	18	ICA	Peak flow velocity (cm/s)	61 (7)	48 (3)	−21

* *Average of two or more non-significant groups*.

† *Mean (95% confidence intervals). Studies in bold indicate those included in the meta-analyses. HRT, hormone replacement therapy; ICA, internal carotid artery; CCA, common carotid artery; ECA, external carotid artery; MCA, middle cerebral artery; PCA, posterior cerebral artery; VA, vertebral artery; ACA, anterior cerebral artery*.

HRT studies (*n* = 5) showed CBF was 3 ± 12% lower during HRT compared to pre-HRT or control groups. All five studies were included in the meta-analysis, with one study reporting outcomes in two groups using different HRT and two studies reporting outcomes in two vessels (total cohorts = 9). No effects were found in CBF between the high and low hormone phase [−2 cm/s (−6.16, 1.54); *p* = 0.24, *I*^2^ = 75.5%].

Eight studies reported CBF was 10 ± 15% lower in the high hormones phase during pregnancy as compared to the low. Four studies examining CBF across the menstrual cycle showed CBF was 3 ± 10% lower in a high hormone phase compared to a low hormone phase. Across different stages of ovarian hyperstimulation (*n* = 2) CBF was 12 ± 7% higher in the high hormone phase compared to the low. Four studies investigating menopause showed CBF was 23 ± 25% higher in the high hormone phase, while oral contraceptive studies (*n* = 2) reported that CBF was 15 ± 4% lower in the high hormone phase as compared to the low.

### Effect of Changing Ovarian Sex Steroid Hormones on Resistance Index

Eleven included studies reported RI in a low and high hormone phase (Table 4). RI was most frequently reported using the ICA (*n* = 7), with fewer studies reporting the MCA (*n* = 5), ECA (*n* = 4), CCA (*n* = 4), PCA (*n* = 1), and VA (*n* = 1).

**Table 4:** Characteristics for included studies reporting Resistance Index (RI).

**Study**	**Technique**	**Hormone group**	**Sample size (n; low hormone phase, high hormone phase)**	**Vessel insonated**	**Outcome measure**	**Low hormone phase; mean (SD)**	**High hormone phase; mean (SD)**	**% change in RI from low to high hormone phase**
**Cagnacci et al. (****2000****)**	Vascular Doppler Ultrasound	HRT	18, 13	ICA	RI	0.66 (0.09)	0.64 (0.05)	−3
**Clapauch et al. (****2007****)**	Vascular Doppler Ultrasound	HRT	9	ICA	RI	0.60 (0.02)	0.56 (0.02)	−7
**Darj et al. (****1999****)**	Vascular Doppler	HRT	20	CCA	RI	0.75 (0.08)	0.74 (0.06)	−1
	Ultrasound			ECA	RI	0.81 (0.98)	0.80 (0.06)	−1
				ICA	RI	0.59 (0.09)	0.63 (0.10)	7
**Pan et al. (****2002****)**	Vascular Doppler	HRT	40	MCA	RI	0.56 (0.04)	0.57 (0.05)	2
	Ultrasound			CCA	RI	0.69 (0.06)	0.70 (0.07)	1
				ICA	RI	0.58 (0.07)	0.60 (0.08)	3
Brackley et al. (1999)^‡^	Transcranial Doppler Ultrasound	Menstrual cycle	19	MCA	RI	0.55 (0.51–0.59)	0.56 (0.53–0.59)	8
	Vascular Doppler	Menstrual cycle	19	ICA	RI	0.51 (0.49–0.55)	0.54 (0.49–0.58)	2
	Ultrasound			ECA	RI	0.87 (0.83–0.90)	0.86 (0.84–0.90)	−1
Krejza et al. (2003)^•^	Vascular Doppler	Menstrual cycle	14	ECA	RI	0.81 (0.81)	0.84 (0.84)	4
	Ultrasound			ICA	RI	0.60 (0.62)	0.59 (0.58)	−2
				CCA	RI	0.75 (0.74)	0.75 (0.76)	0
Hazlett and Edgell (2018)	Transcranial	Menstrual cycle	14	MCA	RI	0.51 (0.02)	0.52 (0.01)	2
Janzarik et al. (2014)	Transcranial Doppler	Pregnancy	26, 61	MCA	RI	0.46 (0.04)	0.54 (0.06)	18
				PCA	RI	0.43 (0.04)	0.52 (0.06)	21
van Veen et al. (2016)	Transcranial Doppler	Pregnancy	18, 50	MCA	RI	0.43 (0.05)	0.42 (0.04)	−2
Nevo et al. (2007)	Vascular Doppler Ultrasound	Ovarian hyperstimulation	12	ICA	RI	0.14 (0.01)	0.10 (0.01)	−29

*Average of two or more non-significant groups*.

‡ *Median (IQR)*.

• *Mean (median). Studies in bold indicate those included in the meta-analyses. HRT, hormone replacement therapy; ICA, internal carotid artery; CCA, common carotid artery; ECA, external carotid artery; MCA, middle cerebral artery; PCA, posterior cerebral artery; VA, vertebral artery*.

Five studies reported RI with HRT was 1 ± 3% lower in the high hormone phase compared to the low hormone phase. Of these studies, four were suitable for inclusion in the meta-analysis with two studies reporting outcomes in three vessels, resulting in eight cohorts in total. No effects were found in RI between a high and low hormone phase [−0.00 (−0.02, 0.02); *p* = 0.77, *I*^2^ = 57.5%].

RI was 2 ± 3% and 12 ± 13% higher in the high hormone phase when looking across menstrual cycle (*n* = 3) and pregnancy (*n* = 2), respectively. One study investigated changes in RI with ovarian hyperstimulation, reporting a 29% lower RI during high hormone phases compared to low.

### Effect of Changing Ovarian Sex Steroid Hormones on Cerebrovascular Reactivity

Four studies report a measure of CVR within the MCA (Table 5). Two studies investigated CVR using the breath-holding index, one reporting CVR was 79% higher pre-menopause compared to post-menopause, and the other reporting CVR was 32% higher in the high hormone phase of the menstrual cycle compared to the low.

**Table 5:** Characteristics for included studies reporting cerebrovascular reactivity (CVR).

**Study**	**Technique**	**Hormone group**	**Sample size (n; low hormone phase, high hormone phase)**	**Vessel insonated**	**Outcome measure (units)**	**Low hormone phase; mean (SD)**	**High hormone phase; mean (SD)**	**% change in CVR from low to high hormone phase**
Brislane et al. (2020)	Transcranial Doppler/CO_2_ inhalation	Menopause	50, 50	MCA	Absolute change in MCAv per mmHg (cm/s/mmHg)	3.5 (1.9)	3.8 (1.5)	7
Matteis et al. (1998)	Transcranial Doppler/Breath-holding	Menopause	40, 45	MCA	BHI (AU)	0.9 (0.3)	1.6 (0.3)	79
Sherman et al. (2002)	Transcranial Doppler/CO_2_ inhalation	Pregnancy	30, 30	MCA	% change MCAv per kPa (%)	28.0 (6.4)	27.7 (7.7)	−1
Diomedi et al. (2001)	Transcranial Doppler/Breath-holding	Menstrual cycle	20	MCA	BHI (AU)	1.3 (0.3)	1.7 (0.3)	32

*MCA, middle cerebral artery; AU, arbitrary units*.

Two studies examined CVR using CO_2_ inhalation. In pregnancy, CVR (using a target end-tidal CO_2_ 1 kPa above baseline with O_2_ enriched air) was 1% lower in the high hormone phase as compared to the low. Comparing pre- to post-menopausal women, CVR (using a 5% CO_2_, 21% O_2_, balanced N_2_ gas mix) was 7% higher in pre-menopausal women.

### Effect of Changing Ovarian Sex Steroid Hormones on Cerebral Autoregulation

Four studies reported CA measures (Table 6). Two studies investigated CA changes of the MCA in pregnancy, with one study reporting “strength of autoregulation” from the transient hyperaemic response was 7% higher in the high hormone phase compared to the low. The second study in pregnancy reported the autoregulation index at rest as 25% higher in the high hormone phase. One study reported CA changes across the menstrual cycle in the MCA and ACA, showing the autoregulation index during repeated sit-to-stand manoeuvres was 12 ± 3% lower in the high hormone phase compared to low. Finally, one study reported CA using transfer function analysis of repeated squat-to-stand manoeuvres. Comparing pre- to post-menopausal women, phase was 5% lower and normalised gain was 4% higher in pre-menopausal women, indicating marginally less efficient CA in the high hormone phase.

**Table 6:** Characteristics for included studies reporting cerebral autoregulation.

**Study**	**Technique**	**Hormone group**	**Sample size (n; low hormone phase, high hormone phase)**	**Vessel insonated**	**Outcome measure (units)**	**Low hormone phase; mean (SD)**	**High hormone phase; mean (SD)**	**% change in autoregulation from low to high hormone phase**
Favre and Serrador (2019)	Transcranial Doppler/ Sit-to-Stand	Menstrual cycle	13	MCA ACA	ARI (AU) ARI (AU)	3 (1.4) 3.0 (1.2)	2.8 (0.8) 2.6 (1.1)	−10 −13
Sherman et al. (2002)	Transcranial Doppler/Transient Hyperaemic Response	Pregnancy	30, 30	MCA	Strength of Autoregulation (AU)	1.1 (0.2)	1.2 (0.2)	7
van Veen et al. (2016)	Transcranial Doppler/Resting	Pregnancy	18, 50	MCA	ARI (AU)	5.3 (1.4)	6.6 (0.9)	25
Brislane et al. (2020)	Transcranial Doppler/	Menopause	50, 50	MCA	Normalised gain (%)	1.3 (0.4)	1.4 (0.4)	4
	Squat-to-Stand				Phase (degrees)	23.8 (13.2)	22.6 (14.8)	−5
					Coherence (AU)	0.7 (0.1)	0.6 (0.1)	−8

*MCA, middle cerebral artery; ACA, anterior cerebral artery; ARI, autoregulation index; AU, arbitrary index*.

## Discussion

### General Findings

To the best of our knowledge, this is the first systematic review and meta-analysis to examine the interaction between brain vascular function and changing ovarian sex steroid hormones. This review identifies the current gaps in the literature and provides a clear basis from which future research can be established. Most commonly, included studies examined the effects of HRT or pregnancy using either PI or CBF as a measure of brain vascular function. The main outcome from the meta-analyses was that women undergoing HRT had a significant reduction in PI compared to post-menopausal women not on HRT. However, CBF and RI were found to have no changes with HRT.

The following discussion will assess the outcome measures examined in this review in the context of cerebrovascular health and function across the different hormone groups reported.

### Brain Vascular Function and Changing Ovarian Sex Steroid Hormones

The HRT-PI meta-analysis indicated that administration of HRT improved (i.e., lowered) PI in post-menopausal women when compared to post-menopausal women not receiving HRT. This is consistent with previous studies that have shown increases in PI positively correlate with time elapsed since menopause in women not on HRT (Crook et al., 1991). Further, timing of HRT initiation post-menopause has been shown to reduce adverse cardiac events and mortality (Nudy et al., 2019). The majority of included HRT studies in this review report the effects of HRT initiation within 5–10 years of menopause onset (Appendix 3). This “early initiation” may contribute to the beneficial effect of HRT on PI reported herein. However, the considerable heterogeneity in this data set could not be accounted for with meta-regression analysis of potential moderators (type of HRT and insonated vessels), indicating there are unaccounted moderators causing this large variability (discussed below). Limited PI data for the other female hormone groups included in this review determined that a meta-analysis could not be performed. Data from the wider cohort included within this review reported both an increase and decrease in PI from a low to high hormone phase, highlighting inconsistencies within the literature for this measure. PI is used as an indication of downstream resistance to flow, with a higher PI indicative of greater downstream resistance and therefore, reduced cerebral tissue perfusion. A higher PI has been associated with Alzheimer's Disease (Roher et al., 2011), small-vessel ischaemic disease (Kidwell et al., 2001), and poorer post-stroke functional outcomes (Aoki et al., 2013). Thus, it appears that administration of HRT has the potential to alleviate the risk of cerebrovascular disease in post-menopausal women. It could be expected that PI would also be improved in other high hormone phases (i.e., 3rd trimester of pregnancy) but there is insufficient evidence to support this.

The HRT-CBF meta-analysis indicated no overall effects for CBF in post-menopausal women receiving HRT compared to those who were not. As with PI, all hormone groups showed no clear directional change in CBF with changing ovarian sex steroid hormone levels. Since CBF is used as a functional index of cerebral perfusion, CBF could be expected to be greater during high hormone phases. Given that a decline in CBF is believed to precede and contribute to the onset of clinical dementia (Ruitenberg et al., 2005), it is important to identify if HRT during menopause might reduce/delay disease onset particularly for women with other risk factors.

RI was most commonly reported in studies investigating HRT, with four of the five studies in this review suitable for inclusion in the meta-analysis. No significant overall effect was found in RI in post-menopausal women receiving HRT. The small study number and small sample sizes within these studies likely contributed to this outcome. As an index of downstream flow impedance, RI could be expected to be greater in women not receiving HRT as observed with PI. RI reported in other hormone groups showed no clear change in RI from a low to high hormone phase.

CVR was on average greater during high hormone phases compared to low hormone phases, though a formal meta-analysis comparison was not possible due to the limited data available. Of the four included studies, three different hormone groups and three different CVR assessment methods were used. These differences likely explain the wide-ranging changes observed in CVR from the low to high hormone phases (range: −1 to 79%). CVR is an indication of the vasodilatory reserve capacity (Hoiland et al., 2011), with a higher CVR associated with improved cerebrovascular function while CVR impairment can predict the risk of stroke in patients with carotid artery occlusion (Webster et al., 1995; Markus and Cullinane, 2001). Therefore, it could be predicted that high hormone phases would elicit greater CVR, however there is insufficient evidence to conclude this.

The four studies that reported CA showed no clear consensus on the effect with changing ovarian sex steroid hormones. Again, this is most likely due to the range of methods employed to assess CA, the different outcome measures used to index CA, and the low number of included studies. CA has previously been shown to be impaired in stroke (Eames et al., 2002), with a more severe impairment associated with poorer functional outcomes post-stroke (Castro et al., 2017). As such, better CA may be expected during high hormone phases (e.g., pre-menopause compared to post-menopause) but the limited literature available to date provides insufficient evidence to support this.

### Causes of Heterogeneity

This meta-analysis showed that there was considerable heterogeneity within HRT studies. However, while both the composition and routine of HRT interventions varied across included studies (detailed in Appendix 3), meta-regression results showed that HRT type or insonated vessel did not account for this heterogeneity. While age is known to influence cerebrovascular function (Peng et al., 2018), studies investigating HRT effects typically controlled for age. Therefore, age is unlikely to have caused this significant heterogeneity.

Overall, given the limitations in the available data, including the limited number of studies and different methodologies used to assess a broad range of cerebrovascular function measures, the considerable heterogeneity for ovarian sex steroid hormones effects might be expected.

### Recommendations for Future Studies

The 45 included studies in this review illustrates the growing body of knowledge in the area of sex hormones and cerebrovascular function. However, the effect of ovarian sex steroid hormones on brain vascular function still remains largely unknown due to the heterogeneity of the available data and/or the limited number of studies. The following recommendations address these concerns. For the true effect of changing ovarian sex steroid hormones on cerebrovascular function to be better understood future studies must report hormone levels as standard. Of the studies included within this review, only 36% (16 out of the 45) reported hormone levels. Thus, it was not possible to formally compare changes in cerebrovascular function to changes in absolute hormone levels, levels which covered a wide range. For example, plasma progesterone levels during the “high hormone phase” of pregnancy (i.e., 2nd−3rd trimester) is 150 ng/ml as compared to the high hormone phase of the menstrual cycle (i.e., luteal phase), which is 25 ng/ml (Elliott, 2001). Reporting of oestrogen and progesterone could help verify the hormonal status of a group (e.g., phase of menstrual cycle) but also account for individual differences in circulating hormones. In turn, this may help understand some of the variation seen in reported cerebrovascular outcome measures and aid comparisons between studies.

In order to help address the considerable heterogeneity observed in HRT studies, future studies should consider time since menopause and/or time on HRT when recruiting participants. Recording and reporting of this information will allow for assessment of its impact as a moderating factor for the effects of HRT on cerebrovascular function. Additionally, further research should examine the importance of timing, and whether cerebrovasculature benefits occur if HRT is initiated closer to the onset of menopause.

This systematic review highlights the major gaps in the current literature. Only two studies in this review looked at the effect of controlled ovarian hyperstimulation. This has implications for women undergoing *in-vitro* fertilisation (IVF) and the effect of supraphysiological changes in ovarian sex steroid hormones on cerebrovascular function. Furthermore, only two studies investigated changes with oral contraceptive use. A recent NHS England report (NHS England, 2017) found 42% of women in England used oral contraceptives, with the total number presumably being higher when including all types of hormonal contraception (e.g., coil, implant). This high rate of hormonal contraceptive use is in stark contrast to the low volume of research examining its effects on cerebrovascular function and indicates a vast under-representation.

The variance in cerebrovascular measure and/or methodology used to date likely contributed to data heterogeneity. There remains no consensus on the optimal methodology to assess cerebrovascular function, a problem across the literature assessing brain vascular health. Additionally, even within a single outcome measure (e.g., CVR) there can be multiple modes of assessment (e.g., CO_2_ inhalation, breath-holding index). In fact, both duration of the CO_2_ stimulus and the timepoint used for analysis has recently been shown to significantly alter CVR values (Burley et al., 2020). The consistent implementation of both methodology and data extraction is vital to producing robust cerebrovascular function measures and improving the subsequent conclusions regarding brain vascular health. For CVR, Burley and colleagues recommended using a CO_2_ inhalation stimulus duration of 3 min and extracting data from the final 30–60 s. Future research should look to implement consistent methodological approaches across all cerebrovascular outcome measures in order for studies to be comparable.

### Limitations

Due to the low number of studies for most hormone groups, statistical analysis of the data was inappropriate. As such, the relative changes from low to high hormone groups reported here should be used to help identify the knowledge gaps in the literature and aid the direction of future research, rather than to infer the specific effect of ovarian sex steroid hormones on cerebrovascular function at this stage.

The majority of included studies in this review were deemed to be of moderate quality and either a moderate or low level of evidence (i.e., cohort or cross-sectional study design) and therefore a moderate risk of bias. In fact, only some HRT studies were categorised as being of a high level of evidence due to the often clinical-based setting of these studies and the research question being frequently suited to a randomised controlled trial design. It is important to consider the differing quality of evidence across hormone groups when interpreting the results of this review.

The substantial heterogeneity reported within hormone groups should also be considered. The meta-regression performed on HRT studies reporting PI and included in the meta-analysis were unable to identify the moderators driving this heterogeneity. Meta-regression could not be performed in those studies reporting CBF and RI due to the low number of included studies.

This review did not exclude studies based on country or location, and as a result confounding factors such as dietary behaviours or cultural lifestyle differences may account for some of the heterogeneity seen. For example, phytoestrogens, evidenced to cause increases in CBF (Kennedy et al., 2010), are of much greater prevalence in the soy-based foods found in Asian diets compared to Western diets (Rietjens et al., 2017).

### Conclusion

This review has shown that HRT has the capacity to improve PI in post-menopausal women, and therefore the potential to improve cerebrovascular function. There remains insufficient evidence to determine if this effect of HRT is reflected in other cerebrovascular outcome measures. The effect of changes in ovarian sex steroid hormones in hormone groups other than HRT remains largely unclear, at least in part due to the substantial heterogeneity in the current literature and considerable under-representation of certain hormone groups in research (e.g., oral contraception). Despite this, this review provides a foundation for future research through the clear identification of gaps in the current literature. Future research in ovarian sex steroid hormones and cerebrovascular function should aim to improve the consistency and generalisability of findings through reporting of hormone levels and implementation of standardised methodology to assess cerebrovascular function.

## Data Availability Statement

The original contributions presented in the study are included in the article/Supplementary Material, further inquiries can be directed to the corresponding author.

## Author Contributions

Conceptualisation and study design were carried out by BS, SL, and RL. Literature searches and data analysis were carried out by BS, RD, and SW. All authors contributed to the drafting and critical revision of the work.

## Conflict of Interest

The authors declare that the research was conducted in the absence of any commercial or financial relationships that could be construed as a potential conflict of interest.
