# Supplementary material for: A Systematic Review and Meta-Analysis Examining Whether Changing Ovarian Sex Steroid Hormone Levels Influence Cerebrovascular Function
Source: Front Physiol. 2021 Jun 17;12:687591. doi: 10.3389/fphys.2021.687591 (PMC8248489; doi:10.3389/fphys.2021.687591)
Supplement: Supplementary file 1 [file Table_1.DOCX]

|  | **Medline** | **Web of Science** | **Embase** |
| --- | --- | --- | --- |
| 1 | (Cerebrovascular Circulation or Cerebral Arter* OR Middle Cerebral Artery OR Posterior Cerebral Artery OR Carotid Artery, Internal OR Carotid Artery, External).ti,ab,kw | TS=("Cerebrovascular Circulation" OR "Cerebral Arter*" OR "Middle Cerebral Artery" OR "Posterior Cerebral Artery" OR "Carotid Artery, Internal" OR "Carotid Artery, External") | ('brain circulation' or 'brain blood flow' or 'brain artery' or 'posterior cerebral artery' or 'middle cerebral artery' or 'external carotid artery' or 'internal carotid artery').ti,ab,kw. |
| 2 | (Gonadal Steroid Hormones or sex or menstrual cycle or follicular phase or luteal phase or ovulat* or estradiol or estradiol congeners or progesterone congeners or luteinizing hormone or follicle stimulating hormone or premenopause or perimenopause or postmenopause or menopaus* or hormone replacement therapy or Fertilization in Vitro or Ovulation Induction or ovulation inhibition or contraceptive agents, female or Contraceptives, Oral, Hormonal or pregnancy).ti,ab,kw | TS=("Gonadal Steroid Hormones" or sex or "menstrual cycle" or "follicular phase" or "luteal phase" or ovulat* or estradiol or "estradiol congeners" or "progesterone congeners" or "luteinizing hormone" or "follicle stimulating hormone" or premenopause or perimenopause or postmenopause or menopaus* or "hormone replacement therapy" or "Fertilization in Vitro" or "Ovulation Induction" or "ovulation inhibition" or "contraceptive agents, female" or "Contraceptives, Oral, Hormonal" or pregnancy) | ('Sex hormone' or sex or 'menstrual cycle' or 'follicular phase' or 'luteal phase' or ovulation or estradiol or progesterone or 'luteinizing hormone' or follitropin or menopause or postmenopause or climacterium or premenopause or 'estrogen therapy' or 'hormone substitution' or 'in vitro fertilization' or pregnancy or 'ovulation induction' or 'ovulation inhibition' or 'hormonal contraception' or 'contraceptive agent').ti,ab,kw. |
| 3 | 1 AND 2 | #2 AND #1 | 1 AND 2 |
| 4 | 3 NOT (fetus or fetal or child* or neonatal).ti,ab,kw | #3 NOT TS=(fetal or fetus or neonatal or child*) AND #3 Not TI=(fetal or fetus or neonatal or child*) | 3 NOT (fetus or fetal or child* or neonatal).ti,ab,kw |
| 5 | 4 NOT (animal* or rat*).ti,kw | #4 NOT TS=(animal* or rat*) AND #4 NOT TI=(animal* or rat*) | 4 NOT (animal* or rat*).ti,kw |

**Appendix 1:** search strategy; conducted 10^th^ March 2021.
