# Supplementary material for: A Systematic Review and Meta-Analysis Examining Whether Changing Ovarian Sex Steroid Hormone Levels Influence Cerebrovascular Function
Source: Front Physiol. 2021 Jun 17;12:687591. doi: 10.3389/fphys.2021.687591 (PMC8248489; doi:10.3389/fphys.2021.687591)
Supplement: Supplementary file 2 [file Table_2.DOCX]

| **Level 1 (High):** | **Level 2 (Moderate):** | **Level 3 (Low):** |
| --- | --- | --- |
| A control group was used | Pre-/post- and/or repeated measures design was used | Post-test only OR cross-sectional design was used |
| A pre-/post- or repeated-measures design was used | A control or comparison group may have been used, but was not required | Case Studies (individual or very small cohort) |
| Groups were randomised | Groups were not required to be randomised | Uncontrolled study |
| Example: Randomised Control Trial | A retrospective design may be used | A retrospective design may be used |
|  | Examples: Cohort, Case-Control, Time Series studies | Example: Cross-sectional study |

**Appendix 2:** Level of evidence criteria for included studies.
