# Supplementary material for: A Systematic Review and Meta-Analysis Examining Whether Changing Ovarian Sex Steroid Hormone Levels Influence Cerebrovascular Function
Source: Front Physiol. 2021 Jun 17;12:687591. doi: 10.3389/fphys.2021.687591 (PMC8248489; doi:10.3389/fphys.2021.687591)
Supplement: Supplementary file 3 [file Table_3.DOCX]

**Appendix 3:** Hormone Replacement Therapy (HRT) characteristics for included HRT studies.

| **Study** | **Study Intervention - HRT type** | **Time on HRT Prior to Study** | **Time Since Menopause** |
| --- | --- | --- | --- |
| Acar et al. (2005) | Intranasal 17β-oestradiol (300μg) | Participants not receiving HRT prior to study | Mean ± SD: 3.6 ± 2.6 years |
| Cacciatore et al. (1998) | Assigned either oral oestradiol (2 mg/d; 12 d/month), combined with oral norethisterone acetate (1 mg/d; 10 d/month) and followed by oestradiol (1 mg/d; 6d/month), or transdermal oestradiol (50μg/d; 14d/month), combined with norethisterone acetate 0.25 mg/g; 14 d/month). | Participants either not receiving HRT prior to study or had a minimum washout period of 2 months. | Range: 6 months - 5 years |
| Cagnacci et al. (2000) | Continuous transdermal HRT (50 mg/day oestradiol, plus 10 mg/day medroxyprogesterone acetate for 12 days every 28 days) | Participants were on HRT for at least 6 months prior to the study. | Range: 1 - 4 years |
| Clapauch et al. (2007) | Intranasal 17β-oestradiol (300μg) | Participants were using either transdermal oestrogen, or transdermal oestrogen with cyclical dihydrogesterone prior to study. Time period not stated. | Mean ± SD: 6.4 ± 3.5 years |
| Crook et al. (1991) | Assigned transdermal oestradiol (50μg/d) for weeks 1-6, combined with medroxyprogesterone acetate (10mg/d; 12d/month) for 12 days a month for weeks 10-21. | Not stated. | Median (range): 24 months (8-96 months) |
| Darj et al. (1999) | Oral 17β-oestradiol (2mg/d; 12d/month), combined with norethisterone acetate (1mg/d; 10d/month), followed by 17β-oestradiol (1mg/d; 6 d/month) | Not stated. | Mean (range): 38.1 months (6 - 108 months) |
| Guvenal et al. (2009) | Assigned to either conjugated equine oestrogens (0.625mg/d) or combined with medroxyprogesterone acetate (2.5 mg/d) | Participants not receiving HRT prior to study. | Not stated. |
| Huang et al. 2009 | 17β-oestradiol (2mg/d) with norethisterone acetate (1mg/d) | Participants not receiving HRT prior to study. | Mean: 1.5 years |
| Jackson & Vyas (1998) | Oral oestradiol (2 mg daily) | Participants either not receiving HRT prior to study or had a minimum washout period of 12 months. | Median (range): 11 (1-28) years |
| Lazar et al. (2004) | Oral oestradiol (2mg/d) and norethisterone acetate (1mg/day) | Participants either not receiving HRT prior to study or had a minimum washout period of 6 months. | Mean ± SD: 6.6 ± 6.1 years |
| Naessen & Bakos (2001) | A 20mg oestrogen implant placed subdermally every 6 months | Participants had an average HRT duration of 18.8 years (range 5.8 –33.9 years) prior to the study. | Not stated. |
| Pan et al. (2002) | Conjugated equine oestrogens (0.625mg/d) combined with medroxyprogesterone acetate (5 mg/d) | Participants either not receiving HRT prior to study or had a minimum washout period of 3 months. | Mean ± SD: 1.8 ± 2.8 years |
| Penotti et al (1996)a | Transdermal 17β-oestradiol (50 μg/d), combined with medroxyprogesterone acetate (10mg/d) for 12 days every two months. | Participants had received HRT for at least 1 year, but less than 2 years prior to the study. | Mean ± SD (range): 4.71 ± 2.03 years (3 - 12 years) |
| Penotti et al. (1998) | Transdermal 17β-oestradiol (50 μg/d) | Not stated. | Not stated. |
| Persico et al. (2005) | Continuous oestradiol transdermal supplementation (50 µg/d) combined with medroxyprogesterone acetate (10 mg/d; 12d every 2 months) | Participants not receiving HRT prior to study. | Mean ± SD (range): 3.3 ± 1.5 years (1–8 years) |
| Vidovic et al. (2001) | Combined oral oestradiol (2mg/d) and norethisterone acetate (1mg/d) | Participants not receiving HRT prior to study. | Minimum of 12 months. |
| Wender et al. (2011) | Assigned to either conjugated equine oestrogens (0.625mg/d) or combined with medroxyprogesterone acetate (2.5 mg/d) | Participants either not receiving HRT prior to study or had a minimum washout period of 6 months. | Mean ± SD: CEE group 5.12 ± 4.95 years; combined CEE+MPA group 5.76 ± 4.29 years |
